# Cell autonomous and non-autonomous consequences of deviations in translation machinery on organism growth and the connecting signalling pathways

**DOI:** 10.1098/rsob.210308

**Published:** 2022-04-27

**Authors:** Agustian Surya, Elif Sarinay-Cenik

**Affiliations:** Department of Molecular Biosciences, University of Texas at Austin, Austin, TX, USA

**Keywords:** ribosomes, ribosomopathy, haploinsufficiency, cell competition, cell non-autonomous

## Abstract

Translation machinery is responsible for the production of cellular proteins; thus, cells devote the majority of their resources to ribosome biogenesis and protein synthesis. Single-copy loss of function in the translation machinery components results in rare ribosomopathy disorders, such as Diamond–Blackfan anaemia in humans and similar developmental defects in various model organisms. Somatic copy number alterations of translation machinery components are also observed in specific tumours. The organism-wide response to haploinsufficient loss-of-function mutations in ribosomal proteins or translation machinery components is complex: variations in translation machinery lead to reduced ribosome biogenesis, protein translation and altered protein homeostasis and cellular signalling pathways. Cells are affected both autonomously and non-autonomously by changes in translation machinery or ribosome biogenesis through cell–cell interactions and secreted hormones. We first briefly introduce the model organisms where mutants or knockdowns of protein synthesis and ribosome biogenesis are characterized. Next, we specifically describe observations in *Caenorhabditis elegans* and *Drosophila melanogaster*, where insufficient protein synthesis in a subset of cells triggers cell non-autonomous growth or apoptosis responses that affect nearby cells and tissues. We then cover the characterized signalling pathways that interact with ribosome biogenesis/protein synthesis machinery with an emphasis on their respective functions during organism development.

## Graphical abstract

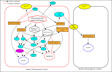

## 1. Variations in the protein synthesis machinery in humans and model organisms

### 1.1. Human pathologies

Germline inheritance and somatic genetic alterations in protein synthesis result in severe developmental congenital disorders and may predispose individuals to cancer [1–7]. Human pathologies resulting from mostly single-copy loss-of-function mutations in ribosome biogenesis (ribosomopathies) and protein synthesis machinery have been reviewed in detail by Venturi & Montanaro [8] and Sarita & Sanal [9]. As an example, **Diamond–Blackfan anaemia (DBA)** encompasses a subclass of diseases called ribosomopathies, which feature a germline-inherited insufficiency in the protein synthesis machinery (specifically in one of the approx. 16 **ribosomal proteins (RPs))**. DBA results in various disorders that have overlapping manifestations, including macrocytic anaemia, congenital defects and a predisposition to malignancy [5,10,11]. DBA is characterized by its unpredictable penetrance and inadequate treatment options [11,12].

Tumour cells often display enlarged nucleoli and have consistently active ribosome biogenesis [13,14]. Paradoxically, several studies have reported high incidences of specific translation machinery mutations in certain cancer types. Among 211 T cell–acute lymphoblastic leukaemia (T-ALL) patients, a subset (11 and 4, respectively) have mutations in *RPL11* and *RPL5*. Paediatric T-ALL patients are particularly affected as overall 10% carry somatic mutations in *RPL10*, *RPL5* and *RPL22* [15]. Strikingly, one particular missense mutation, RPL10 Arg98Ser, was recurrently observed in multiple T-ALL patients [16]. Mutations in *RPL22* have been reported in four out of 47 patients with T-ALL, in 23 out of 30 patients with colorectal cancer and in 17 out of 34 patients with endometrial cancer [17,18]; 22–67% of 7225 cancer specimens from The Cancer Genome Atlas contain deletions of various single RP genes and 12–58% contain deletions in multiple RP genes [19]. Overall, there is not a clear causal link between mutations that affect ribosome biogenesis/function and carcinogenesis. However, cancer cells might have mechanisms to overcome ribosome biogenesis defects by acquiring other mutations. One such example comes from a yeast study, where the authors have modelled recurrently mutated RPL10 Arg98Ser in T-ALL and observed a late 60S biogenesis defect which could be suppressed by another missense mutation (Y37D) in the export factor NMD3. The suppressor mutation in NMD3 rescued the yeast growth defect and resulted in functional RPL10 Arg98Ser containing ribosomes with defects in translational fidelity [20,21]. Decreased translation fidelity could potentially yield more genomic mutations and alterations as a result of proteins that are not correctly translated. Moreover, suppression of different or similar trans-factors could potentially drive carcinogenesis in a similar fashion. As an alternative model, suppressing the overly active translation machinery in cancer cells by ribosome loss-of-function mutations could increase their fitness and survival [15,22].

Patients carrying certain germline inherited mutations in ribosome biogenesis have higher incidences of cancer later in life. Patients with dyskeratosis congenita caused by mutations in *DKC1*, which encodes ribosome biogenesis factor dyskerin, have a higher incidence of cancer, with specifically head and neck squamous carcinoma being the most prominent (approx. 45% cumulative by the age of 50) [23]. In the North American DBA registry of 608 patients for about 15 years of follow-up, 15 patients have developed various solid tumours (such as colon adenosarcoma and breast cancer), two patients have developed acute myeloid lymphoma and two patients have developed myelodysplastic syndromes [24].

Few examples in model organisms suggest that copy number or expression variation in RPs correlate with malignancy. RPL15 overexpression in circulating tumour cells promotes the translation of other RPs and increases lung metastasis in mice [25]. Soft tissue sarcoma was observed in heterozygous *Rpl5* and *Rps24* knockout mice (2 out of 21 and 1 out of 21 animals, respectively) [26]. Heterozygous and homozygous *Rpl22* loss-of-function mutations accelerated the onset and rate of thymic lymphoma progression under the constant expression of the oncogenic gene *MyrAkt* [17,27]. Finally, an unbiased forward genetic screen for tumour suppression across essential genes in zebrafish recovered 12 independent lines with a higher predisposition to peripheral nervous sheet malignancy. Strikingly 11 out of 12 of the screen hits were haploinsufficient for different RP genes [28]. Surprisingly, not much is understood about the mechanism between ribosome biogenesis and cancer. For a detailed reading on this topic, please refer to De Keersmaecker *et al.* [22] and Sulima *et al.* [29].

Recent developments in genomics and pharmacology have provided powerful and novel approaches to first understanding and then intervening in disease-related pathways. While such approaches will be challenging for essential genes with haploinsufficiency conditions, such as DBA or cancer, an interaction ‘roadmap’ can be drawn by investigating which signalling pathways interact with ribosome biogenesis and function in model organisms. Such understanding will ultimately provide an opportunity to understand the underlying disease pathology and rationally develop treatment options for human ribosomopathies (e.g. drugs that interfere with interacting pathways can mitigate DBA-like symptoms in zebrafish and mice) [30,31].

In general, the observed phenotypes in metazoan model organisms encompass developmental delay, the overgrowth of particular tissues and altered apoptosis or cell competition associated with an insufficiency of the protein synthesis machinery, which were further used to screen for factors that would mitigate and suppress such conditions, in order to understand the interacting signalling pathways. A detailed meta-analysis of phenotypic defects caused by multiple RP haploinsufficient mutations in different model organisms is reviewed for further reference [32]. Here, in the next section, we aim to provide a brief overview with examples of these defects in different model organisms.

### 1.2. Budding yeast

Eighty per cent of RP genes are duplicated with functional paralogues owing to a recent genome duplication event in yeast. An interesting study alluded to the paralogue-specific role of RPs where the authors discovered that mutations in genes encoding specific paralogue RP (RPL7, RPL12, RPL13, RPL20, RPL27, RPL34, RPL41, RPP1, RPS4, RPS10, RPS14 and RPS30) mutations had unique effects, ranging from bud site selection to resistance to various drugs. The overexpression of their near-identical paralogues (RPL7B, RPL12A, RPL22B and RPS18A) could not fulfil the same role where deletion of paralogous duplicates of several RPs (RPL7A, RPL12B, RPL22A and RPS18B) affected the budding selection site. These observations suggested a paralogue-specific function of RPs [33]. As the mutants that were used for this study came from the yeast mutant collection, one could imagine that the possibility of fast accumulation of mutations that suppress any growth defects could potentially explain the various observed phenotypes.

To circumvent a potential suppressor mutation accumulation, a more recent study used an inducible degradation system to conditionally prevent ribosome biogenesis in yeast. Multiple ribosomal RNA (rRNA) processing factors (Las1, Rat1, Rrp44, Rrp17) were degraded in an inducible fashion, and the resulting imbalance of newly synthesized orphan RPs was studied. The authors observed that resulting increased orphan RPs triggered a proteotoxic response and resulted in the activation of the conserved heat-shock transcription factor Hsf1 and reduced cellular fitness [34]. Similarly, defects in ribosome assembly induced by depletion of topoisomerase Top1 upregulate Hsf1 targets [35].

### 1.3. Drosophila

Single-copy RP mutations result in a classical phenotype called Minute *Drosophila.* In addition to having a smaller body as a result of their smaller cell size, Minute animals display delayed larval development, short bristles and recessive lethality. Other manifestations of Minute include large and rough eyes as well as reduced viability and fertility [36–41]. Several Minute mutant animals exhibit alterations in the wing structure, weaker legs, paler body colour and chromosome elimination in somatic cells [41,42]. For many decades, the subject of *Minute* loci encoding was unknown until a *Minute* locus (*M*(*3*)*99D*) was successfully linked to an RP-encoding gene, *RpL32* [43]. Over the years, relations between RPs and the Minute phenotype have been well established and extensively reviewed [40,44].

Different gene- or tissue-specific phenotypes have also been observed owing to several RP haploinsufficient mutations. For example, a mutation in the 5′ regulatory region of *RpS6* can induce ectopic cell divisions in haematopoietic organs [45]. A specific Minute phenotype called string of pearls (sop) that is attributed to *RpS2* has been found to manifest in altered ovaries and recessive sterility [46]. Despite the general observation that cell sizes are smaller in *Minute* mutants, larger wing cells and thus larger wings were reported in *RpS13*, *RpS38* and *RpL5* mutants [42,44,47]. These overall results suggest that the loss of function of several RPs might have additional phenotypes that are not easily explainable by overall insufficient protein synthesis.

The general Minute phenotype including body size and short bristles displays non-cumulative and dose-dependent traits. The cumulative effects of different *Minute* alleles are not more severe than the phenotype of a single *Minute* allele, suggesting that the observed phenotype is the result of an overall outcome of insufficiency in the ribosome machinery [41]. When varying levels of RpS3 expression were induced through P-element insertions at different locations in the promoter region, a dose-dependent Minute phenotype was observed; the lower the expression level of RpS3, the more severe the phenotype [48].

Minute or Minute-like phenotypes have also been observed stemming from alterations in other genes related to protein synthesis. First, a well-established *Minute* locus is attributed to a mutation in *eIF2α*, encoding a subunit of the key translation initiation factor eIF2 [44,49]. Second, a phenotype called stubarista, which is attributed to a mutation in a gene that encodes a putative ribosome-associated protein, D-p40, was found to result in shorter antennae, thickened and irregular aristae, short bristles and reduced fertility [50]. Third, several mutations that affect the synthesis of rRNA cause a Minute-like phenotype. A phenotype called bobbed, which affects the locus that encodes 45S ribosomal DNA (rDNA), manifests in smaller bristles and developmental delay owing to the reduced transcription of 45S rDNA [51–53]. Another similar phenotype associated with a reduction in 5S rRNA is called mini (min), and it results in a bobbed-like phenotype and lower viability at non-permissive temperatures [54–56]. Finally, alterations of numerous nucleolar proteins also induce Minute-like phenotypes. *Modulo*, which encodes a DNA-binding nucleolar phosphoprotein, causes a Minute-like phenotype featuring smaller cell sizes and shorter bristles [57–59]. RNAi against *Nopp140*, an evolutionarily conserved nucleolar phosphoprotein C/D box small nucleolar ribonucleoprotein, resulted in delayed development, deformed wings and legs, a higher incidence of short bristles and a degree of lethality [60].

### 1.4. Mouse

Several phenotypes that resemble *Drosophila* Minutes have been observed in mice. A phenotype called belly spot and tail (*Bst*), characterized as the ‘mouse Minute’, is due to a mutation in *Rpl24*. *Bst* animals have kinked tails, white hind feet, skeletal abnormalities and white ventral midline spots. Similar to the Minutes, these animals have smaller body sizes [61]. The mutation of a ribosome-related gene *Rplp1* also results in small body size, male infertility and various systemic tissue abnormalities [62]. While *Rpl29* is not an essential RP gene, its homozygous loss of function results in smaller animals with skeletal defects and embryonic developmental delay [63].

Aside from the developmental delay, various blood disorders and malignancy are associated with RP haploinsufficiency in mice. A series of phenotypes called dark skin (*Dsk*), some of which are attributed to mutations in *Rps19* or *Rps20*, result in increased erythrocyte hypoplasia and pigmentation in the footpads, tails and ears [64]. Homozygous deletion of the *Rpl22* gene results in a P53-dependent defect of *αβ* lineage T cells in the thymus [17]. In an interesting study, the translation of Hox mRNAs was affected in the presence of an RPL38 haploinsufficient mutation, where this mutation drives vertebrate defects. Since this phenotype is largely dependent on mouse genetic background [65] and relies on the haploinsufficient loss of an RP that is conserved in all eukaryotes, the conclusions regarding a gene-specific translation role of RPL38 are debatable [66]. A more recent study found that conserved upstream open reading frames (uORFs) in Hox mRNAs confer alterations in start codon selection stringency and inhibit translation. Depletion of a large RP or using a sublethal concentration of a translation inhibitor can mediate gene-specific effects by altering start codon selection stringency, which argues against sequence-specific, RPL38-dependent translation of Hox mRNAs [67].

### 1.5. Zebrafish

Knockdowns of RPs by morpholinos result in pleiotropic developmental defects [68–71]. Both knockdown and knockout of the *rpl10a* gene resulted in abnormal development, which encompassed short bodies, curved tails and small yolk sac extensions [72]. To model DBA, a knockdown of *rps19* in embryos manifested in defective erythropoiesis, delayed development, shorter body size, a reduced forebrain, defective eyes and death within 10 days post fertilization [73–76]. Single-copy mutations of different RPs cause higher incidences of nerve sheath tumours in zebrafish, which was discovered through a forward genetic screen aimed at finding heterozygous mutations in recessive lethal genes, which suggests that RP genes are potential haploinsufficient tumour suppressors [28].

Both shared and unique zebrafish developmental defects have been observed in knockdowns and mutations in loci that encode several ribosome biogenesis components. These include the snoRNAs U26 (indistinct midbrain–hindbrain boundary, delayed ocular pigmentation), U44 (brain hypoplasia, delayed ocular pigmentation) and U78 (decreased body size, hindbrain defect) [77], *urb2* (digestive organ) [78], *nop10* (bone marrow defects) [79], *nol9* (haematopoietic and pancreatic defects) [80], *wdr3* (craniofacial defects) [81], *esf1* (pharyngeal cartilage, heart, brain and eyes) [82], *nom1* (craniofacial defects and endodermal defects) [83] and *bms1l* (liver) [84].

### 1.6. Caenorhabditis elegans

RP genes are not as extensively studied in *C. elegans* as in *Drosophila*. The mutations and introduction of RNAi against RP genes and protein synthesis machinery components (such as translation initiation) result in larval arrest or developmental delay, increased longevity and reduced fertility in many cases [85]. Targeted double-copy loss of function mutations in five different RPs (*rpl-5*, *rps-23*, *rpl-33*, *rps-30* and *rps-10*) and deletion of repeated 45S rDNA loci in *C. elegans* result in fully completed embryogenesis with no observable defects in embryonically born cells including specialized cell types such as neurons. Thus, the maternally deposited ribosome pool is sufficient for embryonic development. This observation argues that tissue-specific defects mediated by RP haploinsufficient mutations are not likely to be due to the potential sequence-specific translation of certain mRNAs [86]. Intriguingly, a hypomorph mutation in the RNA polymerase I subunit *rpoa-2* (*op259*) in *C. elegans* resulted in increased resistance to ionizing radiation-induced apoptosis in the germline which could be rescued by gain-of-function mutations in Ras/mitogen-activated protein kinase (MAPK) pathways. This observation suggests a genetic link between RNA polymerase I and the MAPK pathways [87].

## 2. Why are certain tissues more affected by the imbalances in subunits of a ubiquitously expressed protein synthesis machinery?

The ribosome is a ubiquitously expressed machinery; thus, one would expect all systems of an organism to be affected similarly. However, certain tissues are more affected by RP haploinsufficiency than others—in both humans and model organisms. For instance, DBA results in severe erythropoiesis defects [5,70,76,88]. Similarly, in mouse haploinsufficient models, blood tissue has been found to be significantly affected [89,90]. Why are certain tissues more affected in response to variations in a ubiquitously expressed protein synthesis machinery? There are three possible explanations for such tissue-specific effects, which we will briefly define in this section (for a detailed discussion, please see the review by Mills & Green [91]).

First, decreased translation affects certain transcripts more significantly than others [91,92]. For example, the haematopoietic transcription factor GATA1 was reduced at the protein level with *RPS19* shRNA knockdown, while its mRNA level was relatively unchanged. The overexpression of *GATA1* partially rescued the growth of *RPS19* knockdown, suggesting the inefficient translation of the *GATA1* gene as the cause of this phenomenon [93]. Transcript-specific defects can be predicted by using a mathematical model that considers the number of ribosomes and the individual mRNA expression levels to predict the translation rate of a particular mRNA [94]. A careful study confirms this model in a classical DBA case with erythropoiesis defects where a single-copy loss-of-function mutation in the *TSR2* gene, a ribosome biogenesis factor, was detected. In this study, the authors observed that reduced ribosome levels—with constant ribosome composition—selectively impair the translation of a subset of mRNAs which impair lineage commitment of haematopoietic stem and progenitor cells [88].

Second, a ‘specialized ribosome’ model indicates that diverse ribosomes could regulate the translation of specific transcripts in a sequence-specific way through tissue-specific RP or rRNA components [95]. Although this is an attractive model, currently it is highly challenging to test it with haploinsufficient mutations of essential RPs that are well conserved throughout all eukaryotes. Furthermore, careful analyses of the RP components via RNA expression have been unable to identify meaningful or significant differences in the ribosome stoichiometry among human tissues [96]. Such differences in RP composition at the protein level have also not been observed in specialized tissues, such as mouse brain tissues [97].

*Caenorhabditis elegans* embryogenesis serves as a strong genetic model for testing potential tissue-specific functions of potentially diverse ribosomes. During *C. elegans* embryogenesis, there is no overall net growth, and, yet, an incredibly diverse set of tissues emerge from a single embryonic cell. In the homozygous loss-of-function mutations of different RPs (*rps-23*, *rpl5*, *rpl-33*, *rps-10*, *rps-30*) or a full deletion to the 45S rRNA locus, the embryogenesis was found to be completed with no tissue-specific defects. The function and morphology of specialized cell types, such as touch receptor neurons, were not affected. These results suggest that a pool of maternal ribosomes is sufficient for the differentiation of diverse cell types, and, thus, the new synthesis of specialized ribosomes is redundant during embryogenesis in *C. elegans* [86]. In two other organisms where embryogenesis does not require extra mass accumulation—*Drosophila* and *Xenopus*—homozygous mutations of the gene encoding a subunit of RNA polymerase I (*RpI135*) and a near-complete deletion of 45S rDNA repeats (a few repeats left in *Xenopus*) have been found to similarly result in complete embryogenesis [98–100].

Third, decreased protein synthesis or broken ribosome stoichiometry trigger certain signalling pathways that could be differentially active in various tissues. The most well-studied example is the P53 signalling pathway via the stabilization of P53 through free RPL5 and RPL10. These two RPs can interact with MDM2/HDM2, the ubiquitin ligase that mediates P53 degradation [91]. Several other RPs can also directly interact with an E3 ubiquitin ligase protein that mediates P53 degradation [101]. Upon activation, P53 mediates programmed cell death and the termination of the cell cycle [102].

In various model organisms, P53 is required for RP-induced developmental defects. Developmental malformations and haematopoietic disorders in zebrafish attributed to several RP (*rps9*, *rps19*, *rpl11*, *rpl29*) knockdowns and mutants are mediated by P53 [75,103–106]. While the transcript of P53 in zebrafish was not altered with five different individual RP (*rps3a*, *rpl23a*, *rpl36*, *rps7* and *rpl11*) mutations, under ionizing radiation, P53 is destabilized at the protein level [107]. The RP deficiency-induced *Bst* and *Dsk* phenotypes in mice are also suppressed by introducing mutations in *P53* [64,108]. Finally, P53 mediates the suppression of cellular protein synthesis in the presence of a single copy of RpS6 in the mesenchymal tissue, by increasing the transcription of 4E-BP, a translation initiation inhibitor protein [109].

## 3. Cell non-autonomous impact of translation machinery alterations in the development of model organisms

In the *C. elegans* mosaic animals where either the posterior or anterior cell of the two-cell stage embryo is an *RP* null adjacent to a wild-type cell [110] there is complete embryogenesis but they are developmentally arrested at the first-stage larvae. This suggests that the growth of the wild-type lineage is prevented by an organism-wide checkpoint in a cell non-autonomous fashion. Moreover, the observed developmental arrest phenotypes are not rescued by the introduction of hypomorphic mutations of insulin/insulin-like signalling (IIS) components *daf-16* and *daf-18*, suggesting the involvement of a distinct pathway that is likely to be separate from the starvation response or dauer formation in *C. elegans* [86]. Similarly, a hypodermis-specific RNAi knockdown of *rps-11* results in a transient developmental arrest, suggesting the role of cell non-autonomous factors in mediating the growth coordination of *C. elegans* larval development [111].

In *Drosophila*, regional or tissue-specific RNAi against several RPs result in cell non-autonomous growth inhibition. Tissue-specific RNAi against *RpS6* in the prothoracic gland caused a non-autonomous developmental defect by inhibiting the secretion of ecdysone, a dipteran-specific growth hormone [112]. *RpL7* RNAi in the pouch region of the *Drosophila* wing inhibited not only the growth of the pouch cell autonomously but also the notum and hinge portion of the wings. Surprisingly, *RpL7* knockdown in the wing pouch also affected the growth of the eye discs, suggesting a coordinated growth across different organs in a cell non-autonomous fashion [113]. The non-autonomous growth coordination mediated by *Rpl7* RNAi in the wing pouch region was dependent on the activation of Xrp1, an insect clade-specific stress-induced transcription factor, and the consequent synthesis of the insulin-like hormone, Dilp8. Dilp8 acted as an inhibitor of ecdysone, which is responsible for coordinating growth across different tissues (we will discuss the detailed mechanism of Xrp1 in the next section) [114]. Future detailed studies with knockdown of different ribosome biogenesis factors or RPs could address these different possibilities. It is furthermore intriguing to contemplate that perhaps similar non-autonomous growth coordination exists in other clades; this remains to be discovered.

In *Drosophila*, mosaic animals composed of Minute phenotype cells (lacking a single copy of an RP gene) and wild-type cells result in the gradual disappearance of the Minute cell lineages, a phenomenon called ‘cell competition’, which is more thoroughly discussed in the next section [115–117]. The disappearance of the Minute lineage requires close proximity of the prospective loser lineage with the faster growing cell lineage [118].

## 4. How are Minute cells eliminated in the mosaic tissues?

As the protein translation capacity is generally tightly correlated with growth, one explanation for the selective elimination of cells with the *Minute* mutation is the differential growth rates. However, cell competition in the mosaic background is not sufficiently explained solely by the difference between the growth rate of the competing lineages [115,119–121]. Moreover, a plethora of cell-to-cell communication and interaction has been reported to be instrumental in inducing cell competition [116,122–127]. In most cases of *Minute* mutation-mediated cell competition, the decline in the loser cells is mediated through apoptosis and engulfment of loser cells [115,119,128].

Elegant genetic studies on *Drosophila* have discovered some of the cellular marks and pathways that define the Minute/loser lineages in a mosaic background. Overall, Minute cells go through integrated stress response and a complex network of pathways are affected which will be summarized below.

First, the *RpL19 Minute*-induced loser lineage in *Drosophila* expresses a specific form of *flower* (*fwe*), which encodes a conserved calcium channel protein conserved in humans (CACFD1) [125]. The expression of the specific isoform *fwe^Lose^* relative to another isoform, *fwe^Ubi^*, is a hallmark of the apoptosis and decline of the *RpL19* Minute lineage [129]. Second, the prospective loser lineage attributed to *RpS3* mutation produces the secreted matricellular protein SPARC, which functions as a protection against cell competition-induced decline [130].

As minute cells go through apoptosis in a mosaic background, mutations in pro-apoptotic genes can suppress the Minute cell competition. The simultaneous deletion of pro-apoptotic genes *head involution defective (hid)*, *grim* and *reaper(rpr)* enabled *Rpl36* haploinsufficient cells to survive the Minute cell competition, suggesting that apoptosis is an important trigger for the elimination of the RP mutant lineage. The ectopic expression of the non-native apoptotic inhibitor P35 also prevented the competition-induced elimination of the Minute lineage. Finally, RNAi against both *dronc* and *dream* caspases also reduced competition between the Minute lineage and wild-type cells [131].

Cell–cell interactions are crucial in mediating Minute cell competition. Decapentaplegic (Dpp), an orthologue of vertebrate bone morphogenic proteins, modulates the growth rate or the cell–cell interactions which are involved in mediating the elimination of Minute cells. A prospective loser Minute lineage (*M*(*2*)*60E/RpL19*) has reduced vesicular endocytosis of Dpp. Their reduced internalization of Dpp activated the transcriptional repressor brinker (Brk). Activation of Brk prevents cell competition by inactivating the activity of the Dpp pathway and promoting apoptosis through c-Jun N-terminal kinase (JNK) [132,133].

The JNK pathway affects cellular growth by simultaneously promoting apoptosis (through previously mentioned caspases hid and rpr) and promoting growth [134]. The downstream targets of the JNK pathway rpr and Scarface are upregulated in RpS3-induced Minute. Moreover, the expression of the negative regulator of the JNK pathway Puckered (Puc) rescued the Minute lineage [135]. However, the JNK pathway involvement has been controversial; a study found that mutants of JNK pathway components (*misshapen, basket*, *RhoABH* and *jun2*) failed to rescue the cell competition [136].

JNK promotes growth through the Janus kinases/signal transducer and activator of transcription (JAK/STAT) signalling, which plays a role in Minute lineage decline by non-autonomously promoting the growth of the wild-type lineage. The expression of a dominant-negative JAK/STAT receptor Dome inhibited the growth in a Minute cell lineage but not the wild-type lineage [135]. Furthermore, Minute mosaic gut tissue secreted JAK/STAT cytokine, Unpaired-3 (Upd-3), from *Drosophila* gut tissue, which is likely to be involved in further growth of wild-type cells. The introduction of a dominant-negative Upd-3 receptor reduced wild-type cell size in the mosaic tissue. These results overall suggest that Minute gut cells secrete the cytokine Upd-3 that promotes the competitive growth of the wild-type lineage [137]. Upd and Upd-2 cytokines are transcriptionally upregulated in the Minute lineage as well [135].

An emerging proposed mechanism of RP-induced cell competition revolves around *Xrp1*, which encodes a dipteran*-*specific DNA-binding protein*.* It emerged as a suppressor of cell competition from two independent genetic screens [138,139]. *Xrp1* was transcriptionally upregulated in RP mutant cells, and its knockdown prevented the occurrence of cell competition and enhanced the growth of Minute cells autonomously [140]. Xrp1 mediates cell competition in Minute cells as a heterodimer with Irbp18, a homologue of the conserved C/EBP binding protein that is essential for double-strand break DNA repair [140,141]. Xrp1 transcriptionally upregulated the pro-apoptotic genes *hid* and *rpr* and the NF-κB orthologue *Dif* (*dorsal-related immunity factor*), which suggests the link between this pathway and apoptosis [139]. P53 is known to mediate cell competition in mammals, and there may be a relation between P53 and Xrp1 in terms of the cell competition in *Drosophila* [142].

Activated Xrp1 also activated the cellular stress response through CncC (Nrf2 orthologue). Paradoxically, the mild activation of the Nrf2 oxidative stress pathway acts as a protective mechanism of the Minute lineage but is sufficient to induce Minute decline upon its over-activation. Both RNAi against and overexpression of CncC increase Minute cell death [135]. RNAi against *Xrp1* in the RpS3 Minute phenotype downregulated the expression of the transcriptional target of CncC GstD1-GFP, rescued the p62 accumulation and reduced the phosphorylation of eIF2α, suggesting the knockdown of *Xrp1* alleviates the integrated stress response induced by RpS3 Minute mutation. Similarly, overexpression of Xrp1 in wild-type animals upregulates GstD1-GFP and the phosphorylation of eIF2α. Finally, RNAi against *Xrp1* rescues the prospective loser status of a wild-type lineage triggered by mosaic overexpression of an Nrf2 orthologue, suggesting that Nrf2 and Xrp1 affect each other in a feedback loop manner [143].

Interestingly, the growth coordination in the wing region and downstream effects on the eye discs that we have discussed in the previous section are likely to be dependent on RpS12, since the combined knockdown of *RpL7* and *RpS12* RNAi in the wing pouch abolishes the growth inhibition observed in the hinge and notum area of wings as well as the eye discs [113]. Surprisingly, RpS12 haploinsufficient animals do not display the Minute phenotype and the RpS12 haploinsufficient mutant Minute lineage is not eliminated by cell competition [114,138]. The ectopic overexpression of RpS12—but not other RPs—resulted in lower survivability in competition, whereas its knockdown and mutation prevented the competitive elimination of RpL36 and RpS18 haploinsufficient cells in the mosaic background [114]. Increased levels of orphan RpS12 activated Xrp1, and the Xrp1 transcription is upregulated in Minute lineages in mosaic tissue in an RpS12-dependent fashion [144,145]. These overall results suggest that orphan RpS12 may act as an indicator of RP haploinsufficiency, and affect the cellular fitness through the involvement of Xrp1, which further affects the growth development through Dilp8 and ecdysone.

Activation of the Toll pathway plays an instrumental role in the competition-induced death of the Minute lineage in competition against the wild-type cells. When the Toll pathway is activated, the activated ligand Spatzle (Spz) binds to several Toll receptors, which in turn causes the phosphorylation and eventual degradation of Cactus (Cact). Under basal conditions, cytoplasmic Cact sequesters the transcription factors Dorsal (dl) (for the developmental programme) and Dif (for the immunity programme). Thus, Dif and dl are translocated to the nucleus upon Cact degradation [146]. RNAi against *dl* and *Dif* or overexpression of Cact rescued the decline of RpL14-induced cell competition [147]. This pathway may be responsible for Minute-induced apoptosis since activation of dl and Dif resulted in the elevated expression of the pro-apoptotic mediator rpr [148]. The pro-apoptotic Salvador–Warts–Hippo pathway is activated by Spz-Toll and it promotes the downstream activity of the Toll pathway [149]. Furthermore, the Salvador–Warts–Hippo pathway has been linked to Minute-induced cell competition since mutations of this pathway's components (*salvador*, *hippo*, *warts*) prevented Minute-induced cell competition [136].

As *Minute* mutations generally affect a single RP gene, the incorporation of other expressed RPs into ribosomes will be reduced owing to the Minute mutation. Thus, Minute mutations would result in higher levels of orphan RPs [150]. Unsurprisingly, proteotoxicity conferred by orphan RPs plays a role in the Minute lineage decline. A proteasome inhibitor bortezomib bolstered the decline of the Minute lineage without affecting the wild-type lineage, while the Minute lineage decline was rescued by alleviating proteotoxicity through rapamycin-induced dTORC1 (*Drosophila* target of rapamycin (TOR) complex 1) inhibition and overexpression of the conserved transcription factor FOXO1 [121]. Similarly, another study found that two RP-induced cell competitions (*RpS23*^R67 K/+^ and *RpS26^KO/+^*) induced proteotoxic markers (phosphorylated eIF2α) and apoptosis, triggered by Xrp1 and inactivated dTORC1 pathway [120].

**Figure 1.**
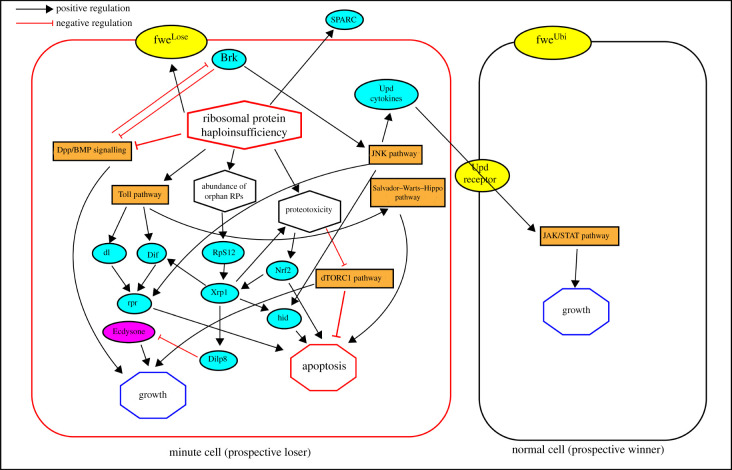
Minute phenotype results in ‘cell competition’ where prospective Minute cells are eliminated in a wild-type mosaic background through activation of several signalling pathways. The activated pathways as summarized in the chart reduce growth and/or promote apoptosis of the prospective loser Minute lineage. Concomitantly, the decline of the Minute lineage is exacerbated by the non-autonomous growth promotion signalled by the Minute lineage itself.

In conclusion, Minute-induced cell competition is mediated through various intertwining pathways that result in an integrated stress response that eventually leads to either decreased growth or increased apoptosis of the Minute lineage. We summarize the mechanisms that we have discussed in this section in figure 1.

## 5. Signalling pathways that regulate ribosome biogenesis and protein synthesis

In this section, we summarize through which mechanisms MYC transcription factor, TOR and RAS/ERK/MAPK signalling pathways regulate both ribosome biogenesis and protein synthesis. In addition, we briefly cover the phenotypes associated with alterations of these pathways in different organisms. For further reading and learning of other signalling pathways that interact with ribosome biogenesis and protein synthesis, please refer to Simpson *et al*. [151] and Song *et al*. [152].

Ribosome biogenesis is an energetically costly process that requires careful coordination between all RNA polymerases and a plethora of assembly factors. Ribosome biogenesis involves all the RNA polymerases: RNA Pol I for the synthesis of 47S pre-rRNA, RNA Pol II for the synthesis of the RPs as well as the assembly factors and small nucleolar(sno-) RNAs and RNA Pol III for the synthesis of pre-5S rRNA and tRNA [153].

Protein synthesis involves distinct initiation, elongation and termination steps. Translation initiation begins with the formation of the 43S preinitiation complex containing the 40S ribosome unit, followed by mRNA activation, 43S binding to mRNA, mRNA ribosome scanning, the initiation of codon recognition and recruitment of the 60S ribosome subunit. These processes were mediated by multiple eukaryotic initiation factors [154]. Translation elongation involves the binding of aminoacyl-tRNA at the A site of the translating ribosome, the formation of a peptide bond and translocation. These steps feature the roles of several eukaryotic elongation factors [155]. Termination occurs when the translating ribosomes recognize termination codons at the A site, which promotes the hydrolysis of peptidyl-tRNA on the P site, and finally the release of the nascent peptide [156]. Among these three steps of protein synthesis, the rate-limiting step comprises the translation initiation [157].

**Figure 2.**
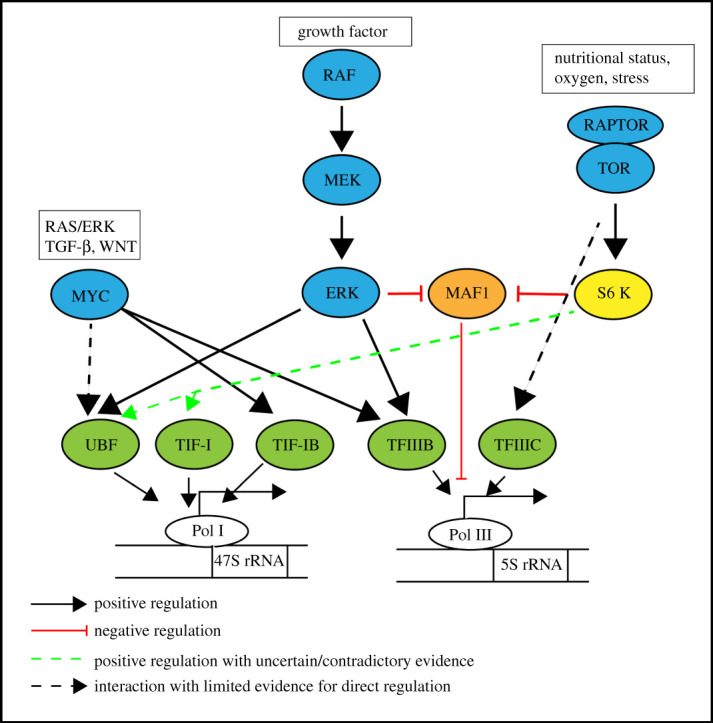
Environmental and cellular factors regulate ribosomal RNA (rRNA) transcription through MYC, RAS/ERK and TORC1. MYC interacts directly with factors involved in the transcription of ribosomal RNA precursors to promote the transcription of ribosomal RNAs Ras/ERK and TORC1 pathways activate the ribosomal RNA precursor transcription through phosphorylation of the transcriptional factors. All of them work through transcriptional initiation factors of both RNA polymerases I and III, which synthesize 47S rRNA and 5S rRNA, respectively.

**Figure 3.**
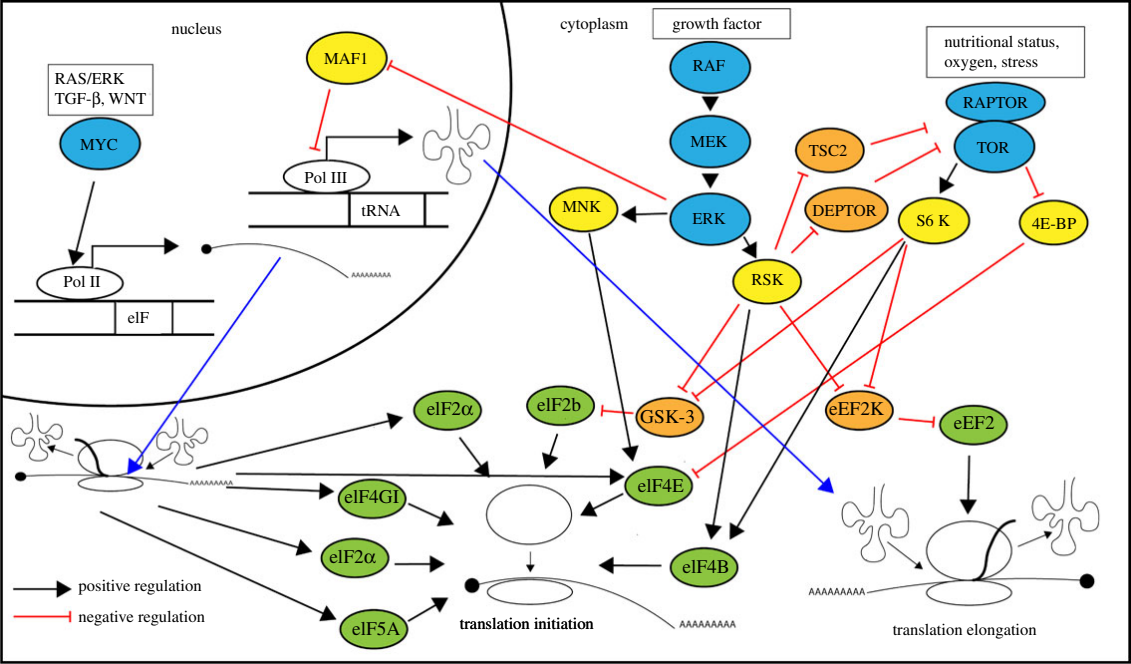
Protein synthesis is regulated through the initiation and elongation steps by MYC, RAS/ERK and TORC1. MYC promotes the Pol II-mediated transcription of protein synthesis machinery components. RAS/ERK and TORC1 positively regulate translation initiation by direct phosphorylation of a series of initiation factors. RAS/ERK and TORC1 pathways phosphorylate eEF2K, which negatively regulates translation elongation.

The 45S rRNA initiation factors and protein translation factors are regulated by the RAS/ERK, mTORC1 and MYC signalling pathways in an intertwined manner. MYC can be stabilized through phosphorylation by RAS/ERK signalling, and the expression of MYC is promoted by mTORC1 through the involvement of both S6 K (RPS6 kinase) and 4E-BP1 via their associations with eIF4B and eIF4E, respectively [158–162]. mTOR and the RAS/ERK signalling pathways upregulate 45S rRNA transcription via the binding of the transcription factors to either the rRNA core promoter region or upstream control elements, which include TIF-IA/Rrn3, selective factor 1 (SL1)/TIF-IB and the upstream binding factor (UBF) [160,163–166]. We summarize how these three pathways affect ribosome biogenesis in figure 2, and their effect on protein translation in figure 3. For a detailed review on this topic, please refer to Kusnadi *et al.* [160] and Goodfellow & Zomerdijk [163].

## 6. The MYC transcription factor

MYC, a conserved sequence-specific transcription factor, is instrumental in cellular growth. MYC promotes the activity of RNA Pol I and RNA Pol III, modulates chromatin and upregulates the RNA Pol II-mediated transcription of protein synthesis and ribosome biogenesis factors. First, MYC has an affinity for the promoters of genes encoding SL1 subunits Rrn3 and UBF [158,167]. The MYC–MAX (MYC-associated factor X) complex regulates Pol I through a preferential binding to the rDNA locus and its association with the SL1 (an orthologue to TIF-IB) complex [158]. Second, MYC binds to the promoter and the terminator of rDNA regions, recruiting GCN5 acetyltransferases and/or Tip60 histone acetyltransferase complexes to promote histone H4 acetylation at the rDNA loci; the resulting chromatin changes increase the transcription activity in the rDNA loci [168,169]. Third, MYC interacts with BRF1, a subunit of TFIIIB [158], which increases the RNA Pol III activity [170]. Fourth, MYC upregulates the transcription of RP genes via an affinity with their promoter in both mice and humans; however, such a phenomenon has not been observed in invertebrates [171]. In *Drosophila*, dMyc does not regulate RP gene transcription [172], and *C. elegans* does not possess a homologue of MYC [173]. Finally, MYC promotes the expression of over 60 nucleolar proteins in murine cell lines, many of which play multiple roles in rRNA processing and ribosome maturation [174].

The role of MYC in protein synthesis is also widely documented. The inactivation of MYC in lymphoma cells suppresses protein synthesis [175]. MYC overexpression results in increased levels of initiation factors eIF2α, eIF4E, eIF4AI and eIF4GI [171,176–179]. The pathways that interact with MYC also feed into protein synthesis and ribosome biogenesis. MYC is regulated by multiple growth signalling pathways, such as WNT, TGF-β and RAS/ERK [180]. ERK can increase the stability of MYC by phosphorylating Ser62 and prevent the degradation of MYC by dephosphorylating Thr58 [181].

Since MYC is so tightly correlated with ribosome biogenesis and protein synthesis, loss-of-function mutations in c-MYC result in small body phenotypes and developmental defects, such as smaller wings and slender bristles in *Drosophila*, as well as heart defects in c-MYC knockout mice [173,182]. Expectedly, the overexpression of dMyc elevates the cell size and cellular growth rate in *Drosophila* [183]. However, the hypomorphic loss-of-function mutation in mouse MYC (c-MYC) does not lead to an observable change in cell size, suggesting the involvement of other pathways in the regulation of cell size [184,185].

## 7. TOR pathways

The TOR pathway takes the input of the cellular nutrition status to affect all steps of ribosome biogenesis and protein synthesis, and this pathway is highly conserved from yeast to human models [186–189]. TORs (mTOR in mammals, dTOR in *Drosophila*) are conserved serine–threonine kinases that only function as complexes. TOR complex (TORC) was originally discovered as the mediator of the immunosuppressive effect of rapamycin [190]. There are two different types of TORCs: which are TORC1 and TORC2. TORC1 is susceptible to rapamycin, while TORC2 is not [180]. The catalytic activity site of both TORCs is on the TOR protein, but the TORC target specificity is largely determined by its partner proteins—RAPTOR or RICTOR for TORC1 and TORC2, respectively [191].

mTORC1 takes amino acid and energy levels as an input and its activity is related to the increased translation and protein and lipid metabolism as well as the prevention of the breakdown processes encompassing apoptosis, proteasome activity and lysosomes [189]. In yeast, TORC1 mainly senses nutritional input by using a complex consisting of Rag-GTPases/GTR proteins and LAMTOR/EGO proteins. In mammalian cells, the Rag proteins form a complex with the Ragulator complex, and the complex they form serves as an amino acid sensor [190,192]. There are other TOR components: Sestrin2, CASTOR1 and SAMTOR that serve as specific amino acid sensors for leucine, arginine and methionine, respectively [193–196]. AMP-activated kinases (AMPK) are the ‘ATP status sensors’ of the cells, and they regulate TOR signalling as well [197]. The GTPase, Rheb and the kinases AMPK and PKB/Akt all regulate TORC1 activity, connecting the TORC1 pathway with cellular amino acid and energy levels [192].

TORC1 extensively regulates ribosome biogenesis and protein synthesis in multiple ways. First, TORC1 activation results in rDNA amplification [198]. Second, TORC1 directly interacts with the promoters of the rDNA (both pre-47S and 5S) and genes transcribed by RNA Pol I and Pol III. These interactions are inhibited by rapamycin without altering the overall protein levels [199]. A rapamycin-mediated TORC1 inhibition also inhibits pre-rRNA processing and maturation [200]. Third, mTORC1 suppresses the activity of the repressor of RNA Pol III Maf1 via phosphorylation [201]. Fourth, TORC also interacts with TFIIIC, suggesting that TORC1 could also regulate 5S rRNA transcription by affecting RNA Pol III recruitment [202].

The two main direct phosphorylation targets of TORC1 in regulating protein synthesis are RPS6 kinase (S6 K) and eukaryotic initiation factor 4E binding protein (4E-BP). The TORC1 phosphorylation of 4E-BP results in the latter's release from eIF4E, enabling eIF4E to form an initiation complex for translation [203–205]. The other main target of TORC1 is p70 RPS6 kinase (S6 K). TORC1 phosphorylates S6 K, which affects the three main steps of protein synthesis. First, S6 K phosphorylates eIF4B to promote translation initiation [206]. Second, S6 K inhibits the activity of a negative regulator of protein synthesis, eEF2 K [207]. Third, S6 K1 promotes the helicase function of eIF4A by phosphorylating eIF4B on Ser422, since phosphorylated eIF4B can enhance the affinity of eIF4A for ATP [208]. Phosphorylation of RPS6 by S6 K promotes the translation of a set of genes that encode nucleolar proteins involved in ribosome biogenesis [209]. However, the biological role of RPS6 phosphorylation in protein synthesis has been controversial [210,211].

While the ability of TORC1 to influence rDNA transcription initiation is well established, there are conflicting explanations regarding its mechanism in this context. Rapamycin treatment, which inhibits TORC1, alters the phosphorylation pattern of TIF-IA in human cell lines, albeit neither TORC1 nor S6 K directly interacts with TIF-IA [212]. This result was confirmed by a study in yeast, in which rapamycin treatment reduced the association between RNA Pol I and Rrn3 (the yeast homologue of TIF-IA) through the latter's dephosphorylation [213]. However, another yeast study suggested that rapamycin treatment did not inhibit Rrn3, but resulted in dephosphorylation of UBF instead [214]. Thus, TORC1 activates rDNA transcription through activation of Pol I, through phosphorylation of either TIF-1A or UBF.

TORC2 is a rapamycin-insensitive TOR–RICTOR complex that regulates a plethora of pathways. Specifically, TORC2 regulates the AGC kinase family, which includes protein kinase C (PKC-a), serum and glucocorticoid-regulated kinase 1 (SGK-1) and AKT [186,215,216]. SGK-1 regulates ion transport and cell survival; the PI3 K/AKT pathway is essential for growth and metabolism [216,217] and AKT can regulate TORC1, making TORC2 an upstream regulator of mTORC1 [218]. TORC2 directly interacts with ribosomes and mediates the control of plasma membrane homeostasis and fat metabolism, thus it could potentially mediate the coordination of growth through membrane tension signals. However, a genetic interaction between TORC2 and protein translation is not well established. Plasma membrane tension, induced by mechanically stretching the membrane, induces TORC2 activation by re-localizing Slm proteins on the plasma membrane [219], by phosphorylating the two downstream kinases YPK1 and YPK2 [220–222]. TORC2, in turn, regulates plasma membrane composition, polarity and endocytosis by (i) regulating sphingolipid synthesis [219], (ii) negatively regulating Fpk1, which stimulates flippases that translocate amino glycerophospholipids [221,223], and (iii) mediating communication between plasma membrane adapter proteins Sla2, Ent2 and the actin cytoskeleton as well as recruitment of Rvs167, a protein important for vesicle fission during endocytosis [224].

TORC2 is co-sedimented with ribosomes in a sucrose gradient, and RPL26 is co-immunoprecipitated with the TORC2 complex components rictor, mTOR and mSIN1 [225]. Thus, TORC2 could potentially coordinate cellular growth in response to extracellular cues by mediating communication between growth-mediated membrane tension and protein translation.

TORC plays a necessary role in organismal development in various organisms. In *C. elegans*, mutations in TOR-encoding *let-363*, which plays an instrumental role in both TORC1 and TORC2, result in varying degrees of lethality, depending on the severity of the mutation [226]. A unique developmental arrest in *C. elegans* is attributed to a mutation in *elo-5*, which encodes a protein that synthesizes a specific mono-methylated branched fatty acid. Its derivative, glycosylceramide, feeds into and activates the intestinal ceTORC1 [227–229]. The mice limb patterning defect due to single-copy *Rps6* loss can be suppressed by increasing overall protein synthesis by conditional deletion of tuberous sclerosis complex gene (TSC2), which inhibits the TORC1 pathway [109]. Interestingly, a null mutant of RICTOR orthologue (*rict-1*(*ft7*)) and its target *sgk-1* (Ypk1/Ypk2 orthologues) and *akt-1* results in viable animals, with *sgk-1* mutant animals having increased fat storage and decreased body size in *C. elegans*, suggesting a fine-tuning role of TORC2 in growth and metabolism [230]. Mutation in the Rag GTPase homologue *raga-1* reduces body size in the early adult stage [231].

In *Drosophila*, a genetic screen that yields a phenotype with smaller eyes and a reduced head–body size ratio is mapped to a gene that encodes dTOR [232]. Similarly, a homozygous mutation in S6 K, a target of TORC1, results in a high degree of lethality, while the surviving animals are short-lived and have reduced body size [233]. Moreover, a mutation in dTOR results in delayed development and smaller cellular size while also affecting the cell cycle in the G1/S phase through the suppressed expression of cyclin E [234].

In mice, TOR signalling is far more complex [235]. Homozygous mutation in the kinase region of mTOR results in lethality in mice soon after the embryonic implantation, with the trophoblasts and pluripotent inner cellular mass failing to proliferate *in vitro* [236–238]. Moreover, certain components of the TORC2 complex, such as Rictor, are also embryonic lethal in mice, unlike the viable phenotype observed in *C. elegans,* suggesting the role of TORC2 in mammalian embryonic development [239].

## 8. RAS/ERK/MAPK signalling pathway

RAS/ERK/MAPK is a conserved signalling cascade that transmits signals from cell surface receptors. MAPKs involve three-layer signalling from MAPK kinase kinase (RAF) followed by MAPK kinase (MEK), to MAPK (ERK) through a series of phosphorylations in a hierarchical fashion [240,241]. The downstream kinase of this pathway is ERK, which then phosphorylates p90/RSK ribosomal S6 kinases (RSK) and MAPK-interacting kinases (MNKs) [240,241].

RAS/ERK signalling regulates rRNA transcription by direct phosphorylation of TIF-IA, at Ser633 and Ser649, by ERK [242]. Second, ERK also enhances rRNA transcription by phosphorylating the UBF of the RNA Pol I initiation complex at Thr201 and Thr117, resulting in remodelling of the rRNA locus-associated chromatin and continuous transcription elongation in the rDNA locus [243,244].

The downstream proteins of RAS/ERK signalling, MNKs and p90 RPS 6 kinases (RSKs), play regulatory roles in protein synthesis. First, ERK promotes the synthesis of tRNA by activating TFIIIB Brf1 and inhibiting the activity of the RNA Pol III inhibitor Maf1 [245,246]. Second, MNK activates the translation initiation factor eIF4E [247].

The mTOR and RAS pathways are heavily intertwined [248]. RSK negatively regulates mTORC1 through the inhibition of DEPTOR and TSC2 [247]. RAS/ERK and mTOR both phosphorylate multiple similar proteins involved in translation. First, they phosphorylate RPS6 through different kinases: p90 RSK for RAS and p70 S6 K for mTOR, respectively. Second, they both inactivate glycogen synthase kinase-3 (GSK-3), which phosphorylates and deactivates eIF2B [249,250]. Finally, they both phosphorylate eIF4B to promote translation initiation [251] and phosphorylate eEF2 K, which is a kinase that negatively regulates the elongation factor eEF2 [252].

RAS/ERK signalling plays complex and extensive roles in organismal development. The pathway was first discovered in *C. elegans*, specifically playing a role in vulval development. Overall, the developmental defects in *C. elegans* mutants that affect the RAS pathway cause dysregulation of growth coordination, including zygotic lethality, multi-vulva phenotype, gonadal alterations and sterility [253]. In *Drosophila*, the role of RAS/ERK signalling includes the development of bract cells in the legs and correct tissue patterning [254,255]. In *Drosophila*, RAS is necessary for photoreceptor development in the eye, and the lineage that lacks RAS is outcompeted in the eye tissue [256,257].

In summary, the alterations in the RAS, MYC and TOR pathways often lead to widespread changes in organism development. It is currently not straightforward to disentangle the phenotypic role of these pathways solely on protein translation with genetics as mutations of ribosome biogenesis and protein translation components themselves cause cellular unviability with pleiotropic consequences for organism development.

## 9. Concluding remarks and future directions

In this review, we have discussed the relationship between ribosome biogenesis and development in various organisms, especially regarding the non-autonomous nature of the consequences of alterations in translation machinery on development. The perturbations of ribosome biogenesis can be attributed to the various phenotypes across species, as the recurring outcomes of these mutations are reduced body size and developmental delay. Certain mechanisms that mediate these outcomes have been proposed; however, the direct link between ribosome biogenesis and development has not been completely solved.

It is intriguing to contemplate what future studies will reveal regarding the more detailed mechanistic relationship between ribosome biogenesis and development. At this point, the exact mechanistic relationship between haploinsufficient RP mutations and their phenotype is debatable. Although we have a solid understanding of the regulators of ribosome biogenesis and translation, further studies may emerge that draw direct connections to the absence of ribosomal components.

One key question that remains to be explored is how ribosome biogenesis defects restrict development non-autonomously throughout evolution. For example, in *C. elegans* we currently do not know which tissue and which signalling pathway are responsible for growth arrest in wild-type and RP mosaic animals.

The mechanisms that are being studied in model organisms could potentially be transferable to the human context and shed light on the pathophysiology of the genetic diseases attributed to ribosomopathies or the somatic genome copy alterations of translation machinery frequently observed in cancer.

## Data Availability

This article has no additional data.
